# How do women at increased risk of breast cancer make sense of their risk? An interpretative phenomenological analysis

**DOI:** 10.1111/bjhp.12678

**Published:** 2023-07-03

**Authors:** Victoria G. Woof, Lorna McWilliams, Anthony Howell, D. Gareth Evans, David P. French

**Affiliations:** ^1^ University of Manchester Manchester UK; ^2^ The Nightingale Centre, Wythenshawe Hospital Manchester University NHS Foundation Trust Manchester UK

**Keywords:** breast cancer, high risk, interpretative phenomenological analysis, qualitative, risk appraisals, risk communication

## Abstract

**Objectives:**

Offering breast cancer risk prediction for all women of screening age is being considered globally. For women who have received a clinically derived estimate, risk appraisals are often inaccurate. This study aimed to gain an in‐depth understanding of women's lived experiences of receiving an increased breast cancer risk.

**Design:**

One‐to‐one semi‐structured telephone interviews.

**Methods:**

Eight women informed that they were at a 10‐year above‐average (moderate) or high risk in a breast cancer risk study (BC‐Predict) were interviewed about their views on breast cancer, personal breast cancer risk and risk prevention. Interviews lasted between 40 and 70 min. Data were analysed using Interpretative Phenomenological Analysis.

**Results:**

Four themes were generated: (i) *encounters with breast cancer and perceived personal significance*, where the nature of women's lived experiences of others with breast cancer impacted their views on the significance of the disease, (ii) *‘It's random really’: difficulty in seeking causal attributions,* where women encountered contradictions and confusion in attributing causes to breast cancer, (iii) *believing versus identifying with a clinically‐derived breast cancer risk,* where personal risk appraisals and expectations influenced women's ability to internalize their clinically derived risk and pursue preventative action and (iv) *perceived utility of breast cancer risk notification*, where women reflected on the usefulness of knowing their risk.

**Conclusions:**

Providing (numerical) risk estimates appear to have little impact on stable yet internally contradictory beliefs about breast cancer risk. Given this, discussions with healthcare professionals are needed to help women form more accurate appraisals and make informed decisions.

**Statement of contribution**

***What is already known on this subject?***

- Many women provided with a clinically derived numerical breast cancer risk still hold inaccurate risk appraisals.
- Personal risk appraisals do not appear to be considered in probabilistic terms, but are emotionally laden.
- Personal risk appraisals in the general population are less well known than those who attend family history clinics.

***What does this study add?***

- Personal experience of breast cancer in loved ones impacted how women saw risk and their reactions to risk information.
- Women hold contradictions between abstract knowledge of breast cancer risk and concrete experiences which were not congruent.
- Eliciting personal risk appraisals in clinic could help clinicians improve women's understanding and promote more informed decision making.

## INTRODUCTION

Attempts to introduce a risk‐stratified breast‐screening service within breast cancer screening programmes have been taking place globally (Brooks et al., 2021; Esserman, 2017; French et al., 2020; MyPebs, 2020). Such a service would enable access to more frequent screening and preventative medication for those at increased risk (National Institute for Health and Care Excellence (NICE), 2014). Risk‐stratified screening relies upon models for assessing breast cancer risk. These have traditionally focused on family history; however, recently risk estimation has become more complex. For instance, the Tyrer–Cuzick model incorporates known risk factors, including, family history, reproductive and hormonal factors (i.e., age at menarche and HRT use), and Body Mass Index (BMI) to estimate risk (Evans, Astley, et al., 2016). A similar amount of variance in prediction is explained by two further factors: breast density (ratio of fibro‐glandular tissue to fat; Brentnall et al., 2015; Tyrer et al., 2004) and polygenic risk scores (PRS; number of genetic variants related to hereditable breast cancer risk) from saliva DNA (Evans et al., 2019, 2022). When used with a sample of screening age women in the Predicting Risk of Cancer at Screening (PROCAS) study in the UK, the Tyrer–Cuzick model was well calibrated. Approximately 3% of the 57,000 women sampled in PROCAS were identified as high‐risk (≥8% 10‐year risk), with a further 10% at above‐average (moderate) risk (5–7.99% 10‐year risk; Brentnall et al., 2015; Evans et al., 2014; Evans, Donnelly, et al., 2016) and hence would be eligible for additional screening or prevention offers in line with the NICE clinical guidelines for familial breast cancer (NICE, 2014).

The introduction of a risk‐stratified breast screening service has mostly been appraised positively, with most women indicating they would have their risk assessed if offered (Kelley‐Jones et al., 2021; Mbuya‐Bienge et al., 2021; Rainey et al., 2019; Woof et al., 2020). Women also appear to experience few psychological harms, including a lack of anxiety and cancer worry following risk notification (French et al., 2018, 2023). However, there is still limited understanding as to how women in the general population experience and appraise their breast cancer risk following a clinically derived estimate (a risk estimate based on multifactorial risk prediction model). The majority of research exploring breast cancer risk appraisals following notification of a clinically derived estimate has focused on those with a family history or gene mutation carriers. A recent systematic review of qualitative studies with women at increased risk found that personal risk appraisals were often at odds with the numerical estimate provided, with women turning to their degree of family history or relying on comparisons to affected relatives to make sense of their risk (Woof et al., 2022). This indicates that women may prefer to rely on appraisals formed before being provided with clinically derived estimates. Specifically, where estimates do not reflect assumptions, through being seen as inconsistent with family history, trust in the estimate is negatively affected (Bayne et al., 2020; Woof et al., 2022).

Should risk‐stratified breast screening be implemented at population‐level, millions of women in the UK alone will be affected. Yet, understanding breast cancer risk appraisals in this population is still in the early stages of development, meaning it is not clear how these women make sense of an increased risk. An in‐depth insight of how these women understand and appraise their risk is needed. The present research recruited women from the Breast Cancer Predict (BC‐Predict) study which examined the feasibility of providing breast cancer risk in real time as part of the UK's NHS Breast Screening Programme (French et al., 2020). This study provided an opportunity to explore risk perceptions from women who had received an estimate based on the most accurate risk prediction knowledge to date. Therefore the present study aimed to explore how screening‐age women make sense of breast cancer and their increased risk following notification of a clinically derived risk estimate.

## METHODS

### Design

Women who had received a 10‐year above‐average (moderate; for conciseness will now be referred to as above‐average only) or high breast cancer risk result as defined by NICE clinical guidelines were purposively sampled from the BC‐Predict study (French et al., 2020). To achieve a homogenous sample (Smith et al., 2022; Smith & Nizza, 2022), these two risk groups were chosen as both receive the same preventative advice (offer of additional screening and/or preventive medication) in accordance with NICE clinical guidelines (NICE, 2014). Additionally, all women recruited received risk feedback based on all known risk factors, including a PRS and all were encouraged to attend a risk consultation with a trained healthcare professional (HCP), where risk prevention could be discussed.

### Procedure

Women recruited were given a risk estimate based on the Tyrer–Cuzick model, incorporating breast density and a PRS (Evans et al., 2022). Risk feedback was provided via letter with an accompanying information leaflet, which were designed in accordance with feedback from women who participated in the PROCAS study (Gorman et al., 2022). Letters included notification of their risk together with a list of contributing risk factors, factors which prevented their risk from being higher and prevention advice (see Supplementary Material).

Following risk notification, eligible women were sent information about the present study and asked to contact the team if they were interested in participating in a telephone interview to discuss their risk. Telephone interviews were chosen due to their successful use in previous Interpretive Phenomenological Analysis (IPA) studies (Holland et al., 2016) and due to data collection being conducted during the COVID‐19 pandemic. Unlike face‐to‐face interviewing, building rapport via telephone can be challenging. With this in mind, the primary author compiled a list of conversation starters to put the participant at ease. Advantages of telephone interviewing include the opportunity for the researcher to take notes without causing distraction, as well as arranging interviews to suit participants (Sweet, 2002). Semi‐structured interviews enable flexibility using a topic guide. An interview style that facilitates flexibility is essential to gain in‐depth and experiential data, enabling participants a degree of control over topics raised, providing novel insights and raising issues which may not have been thought of originally (Smith et al., 2022).

Data were collected via one‐to‐one semi‐structured telephone interviews. Interviews were conducted by the primary author, a PhD Researcher with experience of qualitative methods (see Supplementary Material for researcher reflexivity statement). Prior to interviewing and following consenting procedures women provided demographic information (see results and Table 1) for an in‐depth understanding of the sample. Details of women's risk status and contributing risk factors were provided by the BC‐Predict study team following consent.

**TABLE 1:** Participants' demographics and clinical characteristics.

Pseudonym	Age	Education	Index of multiple deprivation (IMD) decile^a^	Clinically derived 10‐year risk estimate	Contributing risk factors	Attended a risk consultation?
Ann	62	O'levels/GCSE	7	Above‐average	Age/Family history/Breast density/BMI 31	Yes
Jill	60	A'level/other post‐16 qualifications at college	9	Above‐average	Age/Family history/30 years+ at first live birth	No
Michelle	54	Degree	8	Above‐average	Age/Family history/30 years+ at first live birth/Combined HRT use 5 years+	Yes
Sue	55	Post‐graduate qualification	8	Above‐average	Age/30 years+ at first live birth	No
Lindsey	51	Degree	8	Above‐average	Family history of prostate cancer/30 years+ at first live birth	No
Bev	59	Degree	8	Above‐average	Age/30 years+ at first live birth	No
Yvonne	60	No qualifications	4	Above‐average	Age/Age at menarche 12 or below	No
Dawn	61	Post‐graduate qualification	8	High	Age/Family history/Family history of uterine & ovarian cancer/Age at menarche 12 or below/BMI 39	Yes

^a^ Higher number indicates least deprived.

Interviews began with a broad question about women's experiences of knowing others with breast cancer, followed by questions about breast cancer risk in general and more specifically their personal risk. Example questions included: *In as much detail as you can, can we start by hearing about any breast cancer experiences (i.e., in your family or social circle) you may have had? Can you describe how you feel when you think about breast cancer? Can you describe to me your experience of receiving your risk of developing breast cancer?* Broad questions were followed with probes to gain more in‐depth information. The topic guide was developed by the research team and reviewed by a Public Patient Involvement and Engagement panel, who had experience of receiving a clinically derived breast cancer risk.

Ethical approval was granted by North West – Greater Manchester East Research Ethics Committee (18/NW/0856). All participants provided audio‐recorded informed verbal consent. Interviews were audio‐recorded, transcribed verbatim and pseudonymised. Interviews lasted 40–70 min.

### Data analysis

Data were analysed using IPA (Smith et al., 2022). IPA is an ideographic method, which aims to understand individuals lived experiences by exploring their unique sense making and the meanings they attribute to a specific phenomenon (Smith et al., 2022). IPA was chosen for this study to gain an in‐depth understanding of women's lived experiences of receiving an increased breast cancer risk estimate. IPA begins as a single‐case analysis, with each participant's narrative considered in the context of their lived experiences before cases are brought together to explore similarities and differences. Case‐by‐case analysis began with a total immersion of transcripts. The primary author listened to the audio recordings and read each corresponding transcript in turn. This enabled the author to re‐familiarize herself with women's experiences. Each transcript was formatted electronically into a table with margins either side. The primary author then began making exploratory notes and initial thoughts about the interviewee's accounts, which populated the right‐hand margin. These exploratory notes focused on the author's interpretations of women's experiences and language use, noting contradictions, salient memories and sense making. These formed the basis of experiential statements, which populated the left‐hand margin. Experiential statements were used to capture the essence of women's thoughts and experiences succinctly. Experiential statements were clustered to form personal experiential themes (PETs). Clustering of experiential statements was carried out by printing the statements and moving them between groupings, which were then colour coded into PETs. PET tables were produced for each interviewee (see Supplementary Material). PETs for each interviewee were then written on post‐it notes and the same process of clustering experiential statements was employed until a final structure of group experiential themes (GETs) was produced. GETs and the theme structure was discussed within the wider team before the final version was developed (see Supplementary Material for the assessment of quality for this IPA study).

## RESULTS

### Participants

Eight women were interviewed, seven above‐average and one high‐risk. All women identified as White‐British, of screening age and had children. Table 1 provides women's pseudonyms and further characteristic details.

### Experiential themes

Four experiential themes were produced: *(i) Encounters with breast cancer and perceived personal significance, (ii) ‘It's random really’: difficulty in seeking causal attributions, (iii) Believing versus identifying with a clinically derived breast cancer risk, and (iv) Perceived utility of breast cancer risk notification*.

#### Encounters with breast cancer and perceived personal significance

Seven women described having known women affected by breast cancer within the family, friendship group or workplace. For Dawn and Michelle breast cancer was described as a defining feature of family life, with Dawn's mother and Michelle's grandmother and aunt affected. Although describing traumatic memories of her mother's breast cancer (who died from the disease), Dawn's worry about a diagnosis diminished over time. She appeared to reach a peak in her concern when she approached the age at which her mother died, with passing this age having protective value. However, concern was transferred to her daughter, indicating the strong association Dawn still holds between the age at which her mother died and the threat of the illness for other female family members:

When I approached 57, my mum died at 57. I mean my brother was the same to be honest, 57 was a terrible year for us […] So, I was very nervous, you're very nervy about it as well and how awful it was and how horrible it was. And I'm also worried about my daughter as well and she's got a daughter now…And it does, it makes you worry… (Dawn).

For Michelle, breast cancer in her family appeared to be a bonding construct, with female family members sharing the same threat. This led to an openness about breast cancer, whereby experiences and concerns are shared constructively:

I'd just say it was natural, just natural for us to talk about it in the family because it was something that happened to all of us. It happened to auntie and my grandma but the after effects of that happened to all of us (Michelle).

The likelihood of developing breast cancer seemed a natural part of Michelle's life, yet not one she was particularly fearful of, with thoughts of breast cancer not occupying her daily. This may be due to her family's engagement in openly discussing the disease, as well as witnessing successful survival stories. Bev also focussed on those who had successfully overcome breast cancer, viewing her colleague's breast cancer through a positive lens, whereby a diagnosis had not stopped her living her life. For Michelle and Bev, contact with those who had overcome the disease provided evidence that there is hope for a fulfilling life after diagnosis.

The personal impact of breast cancer was reduced for women like Ann and Sue who were either physically distanced from their affected loved‐one or had been shielded from their breast cancer. For Ann, her mother's stoical personality and protective nature seemed to leave her on the peripherals of the disease:

I went to see her and things like that but she seemed to be doing alright on her own […] she just seemed to get on with it because she was always a lady that was never going to let things stop her doing what she wanted to do (Ann).

The distinction between active care duties and simply having an awareness of breast cancer in loved ones appeared to have prevented Ann from drawing any deep significance from her mother's illness, protecting her from the realities of the disease. The age at which Ann experienced her mother's breast cancer was also a factor in how she related to the disease. This can also be seen in Jill's recollection of her aunt's diagnosis. Both women recalled being young adults at the time of their relatives' diagnoses, where caring for a family or focusing on building their lives was prioritized, limiting their capacity to think too deeply about the disease:

My Auntie, she had the cancer, I think we're probably talking 20 years ago now, it's a long, long time ago. So I would imagine I was probably in the thick of my family… (Jill).

Ann and Jill also discussed the significance of the cause of death in affected relatives, which was not attributable to breast cancer. Focusing on this appeared to reduce the significance of breast cancer and perceived threat of the illness. This is unlike Yvonne who witnessed her daughter's friend die of breast cancer, causing alarm that the disease can be life threatening for someone so young. Lindsey was the only woman with no experiences of breast cancer in her immediate social circle, relying on those in the public eye to make sense of the disease. Consequently, she did not appear to think about her susceptibility with any regularity.

It seems direct experiences (or lack of experience) with those affected by breast cancer and the nature of the relationship with these loved‐ones influences the significance attributed to the disease and the impact it has had on these women's lives so far.

#### ‘It's random really’: difficulty in seeking causal attributions

Evaluating breast cancer in others appeared to cause confusion as women endeavoured to find logical and definitive patterns to explain disease onset. This often led to contradictory views as women considered new information and ideas during the interviews. All women interviewed experienced difficulties in attributing the cause of breast cancer to health behaviours. When reflecting on her colleague's diagnosis Bev did not consider her a candidate for breast cancer, causing confusion as to why she was diagnosed:

…she was a relatively clean‐living individual so, you know, if you were going to guess who it would happen to, you wouldn't necessarily guess it would be her (Bev).

This confusion appeared to be caused by Bev's personal understanding of what a ‘typical’ woman with breast cancer ought to be like, describing her as someone who engages in ‘risky behaviours’, that is smoking. Lindsey also considered this when trying to understand the causes of celebrity, Julia Bradbury's breast cancer (The Guardian Newspaper, 2021). For women like Bev and Lindsey there appeared to be no reason in the physical and behavioural sense why breast cancer was diagnosed in these women. Also, for Ann there appeared to be no definitive reason for her mother's diagnosis, especially related to diet and health. This called into question the protective link between positive health behaviours and the predictability of breast cancer:

I think you can do everything, you know, try your best to do everything right and it can still, you know, the outcome can still be the same as somebody doing a lot of things wrong (Ann).

Ann considered however her mother's weight and smoking history as potential contributors, but was not convinced by this explanation. Ann's lack of confidence in this explanation might be attributable to her ‘side‐lines’ position during her mother's breast cancer journey, preventing her from accessing information about her diagnosis. Dawn also held confusion over the link between healthy living and breast cancer, instead relying on ‘genetic factors’ to explain the onset of the disease in others. Yet, when discussing her mother's diagnosis Dawn seemed to view this as an afterthought, less convinced by this explanation and instead attributing her mother's breast cancer to external factors:

She had that to cope with [her husband's alcoholism]. He wasn't easy and I think she'd got all the stress of that. As well, she worked; she had to work as well, so I think it goes back to that added in to be honest. And, you know, it reduced her life expectancy. Yeah. I think there might have been genetic, you know, the genetic factors as well (Dawn).

Attributing her mother's breast cancer to external stresses, as well as appearing unconvinced by a genetic link could likely explain why Dawn's concerns about developing breast cancer reduced when she passed the age at which her mother died. Michelle however did attribute breast cancer to a genetic predisposition, likely informed by familial cancers. However, adopting this view led her to characterize breast cancer as potentially uncontrollable:

If my genes have led me down this path, then all I can do is just be aware if it happens, then by examining myself on a regular basis, surely I would find it a little bit earlier, hopefully, and that something could be done (Michelle).

For Michelle breast cancer was viewed as part of her family's identity, it is perhaps this attribution, and her understanding of a genetic predisposition that has driven her to believe the disease cannot be prevented. Jill and Yvonne also viewed breast cancer as fated or predetermined. Like Michelle, Jill viewed breast cancer as uncontrollable, believing the disease is meant for some but not others. This is evident in how she views the genetic trajectory of cancer in her own family:

Weirdly, the thing about the cancer is that in … with my Auntie, her daughter has also had bowel cancer. My thoughts were always … it was that branch of the family, it seemed to be sad that it did seem to go down that branch of the family, the cancer side of things […] Because my mother didn't have any cancer scares of any sort and I remember thinking that, that maybe it was going down that branch of the tree … (Jill).

For Jill, cancer affects her aunt's (her mother's sister) side, causing her to detach from the threat; choosing to believe cancer is unlikely to be in her life‐plan due to the absence of breast cancer in immediate female relatives (i.e., her mother).

Due to difficulties in defining causal attributions women eventually stopped forcing a convincing explanation. Instead, they supported the idea that breast cancer is random and unpredictable. This belief in particular was adopted by Lindsey. Lindsey's difficulty in understanding Julia Bradbury's diagnosis and her lack of direct experience with someone diagnosed seemed to have led her to prefer the view that there is no concrete explanation for a diagnosis.

Witnessing breast cancer in others appears to be the main reference point for these women when trying to make sense of the disease. This caused confusion as prototypes of *women with breast cancer* did not match physical instances, with some preferring to view a diagnosis as random or pre‐determined.

#### Believing versus identifying with a clinically derived breast cancer risk

How women viewed the personal significance of their breast cancer risk estimates also related to the nature of their experiences with breast cancer and ideas of causal attributions. Although having no reason to disbelieve the estimate nor distrust those who calculated their risk, Bev, Lindsey and Sue were dubious about their above‐average risk, as it was not in line with their personal risk appraisals. Bev's personal appraisal of being at ‘low‐ish risk’ was unaltered, believing she was below population risk:

So, if we've got a scale of one to five and average is three, I might've been two (Bev).

Bev's inaccurate recall or disregard for her calculated risk may stem from her belief that she is not a likely *candidate* for the disease, due to her healthy lifestyle and lack of family history. Despite having difficulties in aligning positive health behaviours with a breast cancer risk and diagnosis, Bev still relied on the perceived protective value of healthy living despite seeing her colleague with similar healthy attributes develop the disease. For Lindsey and Sue notification of above‐average risk caused surprise, confused as to why certain risk factors (i.e., having your first child after the age of 30 years) contributed to increasing their risk. Although believing the estimate, Lindsey and Sue did not identify as being at increased risk. Like Bev, this may be explained by how they described women at increased risk and how features such as a strong family history or leading an unhealthy lifestyle do not fit with how they see themselves, despite overall believing breast cancer to be random:

Interviewer: How alike do you think you are to that person [at increased risk] you described?
Lindsey: Well I think I said before things like about family history, yeah, which I'm not aware of any of that in my family, so I don't think I would be alike in that respect. But then other factors are, wrongly or rightly I think is it to do with things like, you know, eating well and all those things. I think well I don't smoke and I don't drink a lot and I eat relatively well and I do exercise. So I don't think I'm the same in that respect.

These contradictory views about the causes of breast cancer and the protectiveness of healthy living appear to be context dependent. For Ann, Jill and Yvonne, an above‐average risk appeared to make no significant impression, taking the estimate at face‐value with little need for emotional processing. Due to her mother's breast cancer, Ann described expecting to be at increased risk. Likely influenced by her mother's stoical personality, Ann actively chose not to dwell on breast cancer, nor did she feel the need to identify too deeply with her risk status. Yet she actively sought a risk consultation, raising the question why she felt the need to seek further information or advice. Jill simply described her risk as ‘reasonable’. This matter‐of‐fact response could be attributed to her view that breast cancer is pre‐determined, a life trajectory she does not identify with. Not only is this evident in how she described her family history but also in how she recalled having a breast lump examined without fearing a diagnosis:

I don't think I felt I had breast cancer…because I've had loads of traumas in my life. So that didn't seem like something that was for me […] I thought, no, I don't think this is my pathway… (Jill).

Although describing herself as ‘naïve’, Jill's decision to distance herself from the threat of breast cancer has appeared to prevent her from identifying with and considering her risk status too deeply.

Contrastingly, Dawn and Michelle appeared to thoroughly identify with their risk, with both considering risk‐reducing medication. Despite expecting to be at increased risk due to their family histories, both described risk notification as shocking. Physical notification appeared to make the threat of breast cancer real, with time needed for processing and acceptance. Engaging in a more in‐depth appraisal of their risk likely stemmed from their active carer roles and first‐hand experiences of familial breast cancer, influencing the importance attributed to their risk. For Michelle, the offer of risk‐reducing medication brought a seriousness to her risk and led to an unexpected journey, where her risk status had to be thoughtfully addressed:

I felt that it needed the time and the space dedicated to actually place it in my life, that was very important that I did that, that it made me stop, it made me look at it and it made me make some decisions. But now I've made those decisions, that's behind me and I get on with what I'm doing and I carry on with my life (Michelle).

Michelle's reaction to the offer of risk‐reducing medication could be linked to her prior beliefs that breast cancer is potentially non‐preventable. Being presented with information about risk‐reducing medication likely challenged this view meaning engagement in more in‐depth processing was required.

For these women identifying with their increased risk status appeared to be linked to whether their prior risk expectations were met, as well as whether the calculated risk was in line with their personal appraisals of what risk factors are necessary to qualify for ‘increased risk’ status.

#### Perceived utility of breast cancer risk notification

The perceived utility of breast cancer risk notification differed widely. Specifically the degree to which women accepted and identified as being at increased risk appeared to affect their overall perceptions of how useful the process was. For Lindsey written notification of her risk left her feeling helpless as for her no new preventative advice was provided:

…there are things that, you know, can help prevent your risk certainly going any higher. Which is things like maintaining a healthy lifestyle; maintaining a healthy weight; limiting, you know, drinking and, sort of, exercising and stuff. But I kind of think I do those things anyway. So I don't really know what else I could do (Lindsey).

Being unable to identify with her risk and the advice provided appeared to leave Lindsey unable to derive anything useful from the process. Bev's inability to identify with her risk also appeared to affect how she viewed the usefulness of risk notification. Believing herself to be at ‘low‐ish risk’ meant she did not identify as someone who would be appropriate for risk‐reducing medication, completely rejecting this idea. Instead, she viewed her risk as something that did not need to be prioritized, describing it as:

…one risk amongst many that we all face day to day, so not one to necessarily get too hung up about (Bev).

This was also the case for Yvonne, who as a result of believing she was provided a low‐risk estimate did not feel the need to take preventative action.

Despite struggling with the link between positive health behaviours and breast cancer risk reduction, the majority of women identified that risk notification reminded them of the importance of remaining as healthy as possible. For Jill preventative lifestyle advice reaffirmed to her the importance of remaining healthy generally, as well as being vigilant of the signs of breast cancer. Jill's response to this advice is somewhat at odds with her belief that breast cancer is not in her life‐plan, raising the question why this information made an impact. This preventative advice may have shifted her perspective slightly, but perhaps not enough for her to fully identify with being at increased risk and completely disregard her view that she is unlikely to develop the disease.

For Ann preventative lifestyle advice seemed less important, attributing her risk to past behaviours which cannot be changed:

…is there anything in my life that I felt differently, I can do differently. Well that could have been when I was younger when I didn't drink as much and I didn't smoke […] but that's gone now, that's gone I can't go back on that (Ann).

Although admitting that engaging in healthy behaviours could reduce her risk, Ann grappled with how preventative this is when healthy women develop the disease. This view appeared to prevent Ann from considering whether her health behaviours needed to be addressed. Ann's expectation that she would be at increased risk may have also had a bearing on how useful she found the risk notification process, reducing any personal impact the result may have had. Similarly, Ann's conscious decision not to dwell on her breast cancer risk and prioritize her mental well‐being is also a likely contributor to how useful she found her risk.

For Michelle and Dawn, risk notification was useful in aiding their decisions to pursue risk‐reducing medication. For Michelle, the preventative information provided enabled her to reassess her view that breast cancer is non‐preventable, leading to a thorough assessment of preventative medication use. Although breast cancer seemed to be a defining feature of familial life, Michelle eventually rejected the idea of medication due to her inability to continue with HRT, which manages her menopausal symptoms, providing her with quality of life. Nevertheless, risk notification and consideration of familial breast cancers led to her opting for a more natural form of HRT. Similar to Bev, Michelle processed her risk status and risk reduction options within the context of more immediate health concerns, despite breast cancer being a strong feature of her familial identity. This was also the case for Dawn, who although choosing to pursue preventative medication, the threat of breast cancer still did not take priority in her life. Like Bev and Michelle, Dawn viewed her breast cancer risk within the context of other health concerns:

…the rheumatoid is shouting because I'm in pain and I'm struggling to walk and I've, you know, I can't use one hand one day and I can't use this foot and I can't do this and I can't do that. So, the rheumatoid really shouts at me all the time, every day. The breast cancer risk doesn't, but it's there, it doesn't go away (Dawn).

Dawn's pragmatic attitude towards her risk seemed at odds with how she recounted the traumatic impact her mother's breast cancer had on her, suggesting although still raw she does not reflect on these experiences with fear, enabling her to gain perspective on her breast cancer risk and focus on more immediate health concerns.

It appears being able to personally identify with a clinically derived risk status affected how women viewed the usefulness of risk notification and prevention advice. Yet, how women find a place for breast cancer risk in their lives does not appear to be associated with how strongly their risk resonates with them but is instead viewed within the context of other health priorities, enabling them to gain perspective over their risk.

## DISCUSSION

Women demonstrated relatively stable breast cancer risk appraisals, which influenced how they connected with their clinically derived estimates. The nature of experience with breast cancer appeared to have an effect on the significance attributed to the disease, as well as views on personal susceptibility. Making sense of the disease and its causes resulted in contradictions, particularly related to the preventative value of positive health behaviours. Whether women identified with their clinically derived risk also seemed to be informed by their views on causation, their pre‐existing expectations and personal risk appraisals. These issues informed whether women found risk notification useful and whether they sought preventative action. Irrespective of whether women identified with their breast cancer risk, risk was viewed within the context of other health concerns or disease risks, indicating breast cancer is just one health issue among many to consider.

In accordance with previous literature (Bayne et al., 2020), for most of the women, provision of a clinically derived risk estimate and associated prevention advice did not influence changes to personal risk appraisals and beliefs about susceptibility. For the majority, pre‐existing risk appraisals were based on family history, genetic predisposition and health behaviours. However, women encountered difficulties in aligning ideas about positive health behaviours with disease prevention; as seemingly ‘healthy’ women develop breast cancer. These contradictions called into question the value of engaging in positive health behaviours. Yet despite encountering contradictions, some women still advocated for the protective value of positive health behaviours when receiving their clinically derived risks. This indicates that although describing the onset of breast cancer to be random overall, some women still retained firm beliefs that engaging in ‘risky behaviours’ increases the likelihood of developing the disease. And so, despite clear notification of factors contributing to their risk, (i.e., having a first live birth after age 30 and age at menarche) these were mostly discounted or not understood. For those who accepted but did not identify with their risk, notification appeared to challenge pre‐existing appraisals and expectations, resulting in some scepticism.

These findings indicate that women possess different ‘lay theories’ or illness representations of breast cancer and breast cancer risk which may not be internally coherent, causing some to encounter difficulties in identifying with their risk and internalizing new ideas. In line with the principles of the common sense model (CSM) of illness representations (Cameron, 2003; Leventhal et al., 1992, 1997) as well as the mental models approach (Downs et al., 2020; Fischhoff, 2013) it is clear that women's schematic representations of the identity and causes of breast cancer are challenged when witnessing physical instances of ‘healthy’ women with the disease. This causes greater cognitive demand when trying to align information from these experiences with stable lay representations of the illness. These conflicting views could affect whether women choose to engage in health behaviours to reduce their risk. This ambivalence is particularly significant when targeting those at increased risk with evidence‐based weight loss programmes. Furthermore, when existing illness representations are fragmented and underdeveloped, new information can be difficult to integrate (Cameron, 2003; Downs et al., 2020; Fischhoff, 2013; Leventhal et al., 1992, 1997), perhaps providing an explanation for why some women were unable to identify with their risk status. This may also explain why some women chose not to pursue a risk consultation or preventative management, as existing beliefs were unaltered by the provision of risk information. However, this was not the case for one woman interviewed, as notification of risk‐reducing medication challenged their view that breast cancer is non‐preventable, indicating illness representations can adapt when what is learnt is internalized and understood (Cameron, 2003; Downs et al., 2020).

Consistent with the present literature (French et al., 2018, 2023), the notification of a personal breast cancer risk does not appear to have caused any lasting emotional distress. For some women their clinically derived increased risk was unsurprising and simply accepted, particularly for those who did not think about their susceptibility with any deep significance. In line with the CSM, it is likely that risk notification provided in the absence of any pre‐existing concern may have caused a transient change in breast cancer risk representation (Cameron, 2003), resulting in matter‐of‐fact responses, non‐identification with the clinical risk estimate and disinterest in preventative management. For example, in line with previous theorizing (Ferrer et al., 2016; Slovic & Peters, 2006) a clinically derived risk estimate may not have been internalized for some women due to low affective arousal associated with the idea of developing breast cancer, as well as distant exposure to those with the disease. Moreover, the cue adaptive reasoning account (CARA) suggests expected information which fits with pre‐existing views requires little affective or cognitive processing. Conversely, when information is unexpected there is demand for these processes to make sense of the incoming information (Renner, 2004). However, for two of the women interviewed, an increased risk although expected was shocking, requiring significant affective and cognitive energy to process. It would appear here that the principles of the CARA are challenged when pre‐existing risk appraisals are emotionally charged due to significant experiences of the disease.

### Strengths and limitations

An IPA typically seeks to explore the lived experiences of salient life events. For some women receiving a clinically derived risk result did not appear to make any significant impression, perhaps limiting the depth of the analysis. However, unlike a typical thematic analysis this IPA has provided an in‐depth exploration into how individuals develop breast cancer risk appraisals, highlighting the subjectivity of receiving risk information and indicating the types of conversations that could be had in clinical settings. In the original BC‐Predict study information about genetic susceptibility (a PRS) and breast density were provided to all women, but the contribution of these factors to overall risk estimates were not explicitly highlighted but used to generate an overall numerical risk/category. This may account for why most women did not discuss the implications of these factors. Future research is needed in a population who have received notification of these risk factors to explore whether this information is internalized into pre‐existing risk appraisals.

### Practical considerations

When delivering breast cancer risk information, HCPs should consider women's existing risk schema in order to provide more personal and meaningful feedback, as these schema are inextricably linked with the formation of health risk perceptions (Cameron, 2003). Although it is not clear how best to do this, HCPs could tailor their discussions using principles from the Tripartite (TRIRISK) model (Ferrer et al., 2016), where personal ideas regarding perceived susceptibility, affective responses and experiential factors could be discussed. HCPs could then use this understanding of current schema to address misinformation, reinforce accurate knowledge and facilitate the internalization of new concepts (Downs et al., 2020). This would enable more comprehensive communication about how a personal breast cancer risk is arrived upon and subsequently aid decisions about preventative management. For example, it is well documented that the uptake of preventative medication for breast cancer is low (Smith et al., 2016). Similarly, the majority of women offered preventative weight loss programmes do not access these services, despite overall there being a positive relationship between communicated breast cancer risk and uptake and retention (Harvie et al., 2019). Facilitating more accurate risk appraisals could likely enable women to see themselves as being *at increased risk*, potentially altering views about taking medication when ‘well’ and perceived eligibility for weight loss programmes. Future research could examine whether facilitating more informed risk appraisals affects behaviour change. However, when communicating about breast cancer risk it will be important to consider that breast cancer may not be the only disease priority, which may affect preventive decisions and outcomes (Woof et al., 2022). Developing campaigns promoting lesser‐known breast cancer causes and risk factors could also facilitate the processing of new information and further develop the accuracy of illness/health risk representations.

## CONCLUSION

Women's views differ regarding the usefulness of receiving a personalized breast cancer risk, with some not identifying with the label ‘increased risk’ but instead favouring personal risk appraisals based on pre‐existing breast cancer illness representations. It seems pre‐existing illness representations have a significant influence on the formation of breast cancer risk perceptions and appraisals, which are mainly informed by personal experiences and social stimuli. Therefore, current practices of providing numerical risk information to promote behaviour change and informed choices are insufficient to change personal risk appraisals. To provide more effective risk communication, HCPs should discuss women's illness representations and attempt to address misunderstandings, identify knowledge gaps and reinforce existing understandings to provide a more accurate basis on which risk representations and appraisals can be formed.

## AUTHOR CONTRIBUTIONS

**Victoria G. Woof:** Conceptualization; methodology; formal analysis; investigation; writing – original draft; data curation; funding acquisition; writing – review and editing. **Lorna McWilliams:** Conceptualization; methodology; writing – review and editing; supervision; funding acquisition. **Anthony Howell:** Conceptualization; methodology; writing – review and editing; supervision; funding acquisition. **D. Gareth Evans:** Conceptualization; methodology; funding acquisition; writing – review and editing; supervision. **David P. French:** Supervision; conceptualization; methodology; formal analysis; funding acquisition; writing – review and editing.

## CONFLICT OF INTEREST STATEMENT

The authors declare no potential conflicts of interests with respect to the research, authorship and/or publication of this article.

## ETHICS STATEMENT

NHS ethical approval for the BC‐Predict study (PROCAS2) was granted by Harrow Research Ethics Committee (18/LO/0649). NHS ethical approval for the present study was granted by North West – Greater Manchester East Research Ethics Committee (18/NW/0856). All participants provided verbal informed consent which was audio‐recorded.

## Supporting information


Data S1

## Data Availability

The dataset analysed is not publicly available as it may contain information that would compromise participant consent. Contact the corresponding author for more information about data availability.
