# Supplementary material for: How do women at increased risk of breast cancer make sense of their risk? An interpretative phenomenological analysis
Source: Br J Health Psychol. 2023 Jul 3;28(4):1169–84. doi: 10.1111/bjhp.12678 (PMC10947456; doi:10.1111/bjhp.12678)
Supplement: Supplementary file 1 — Data S1 [file BJHP-28-1169-s001.docx]

**Supplementary material**

1. **BC-Predict risk notification letter (above-average (moderate) and high) and information leaflet**

| Dear Ms Tester, |  |
| --- | --- |
| **RE: Breast Cancer (BC)-Predict Study** |  |
| **IMPORTANT: ALL CLEAR after your recent mammogram. This is NOT a recall.** |  |

Thank-you for taking part in the BC-Predict study. As part of that study we asked you to complete a questionnaire before you attended your mammogram. In this questionnaire you gave us information which enabled us to work out your risk of developing breast cancer in the next 10 years. Your risk is calculated from a combination of factors associated with your family history, lifestyle and breast density (the amount of tissue in your breast that is not fat).

Your risk of developing breast cancer in the next 10 years was calculated to be **above average (moderate) risk.** This means that 5 - 7% of women in your risk category will develop breast cancer **within the next 10 years.**

The table below provides you with information about your risk category and where you are in relation to other women:


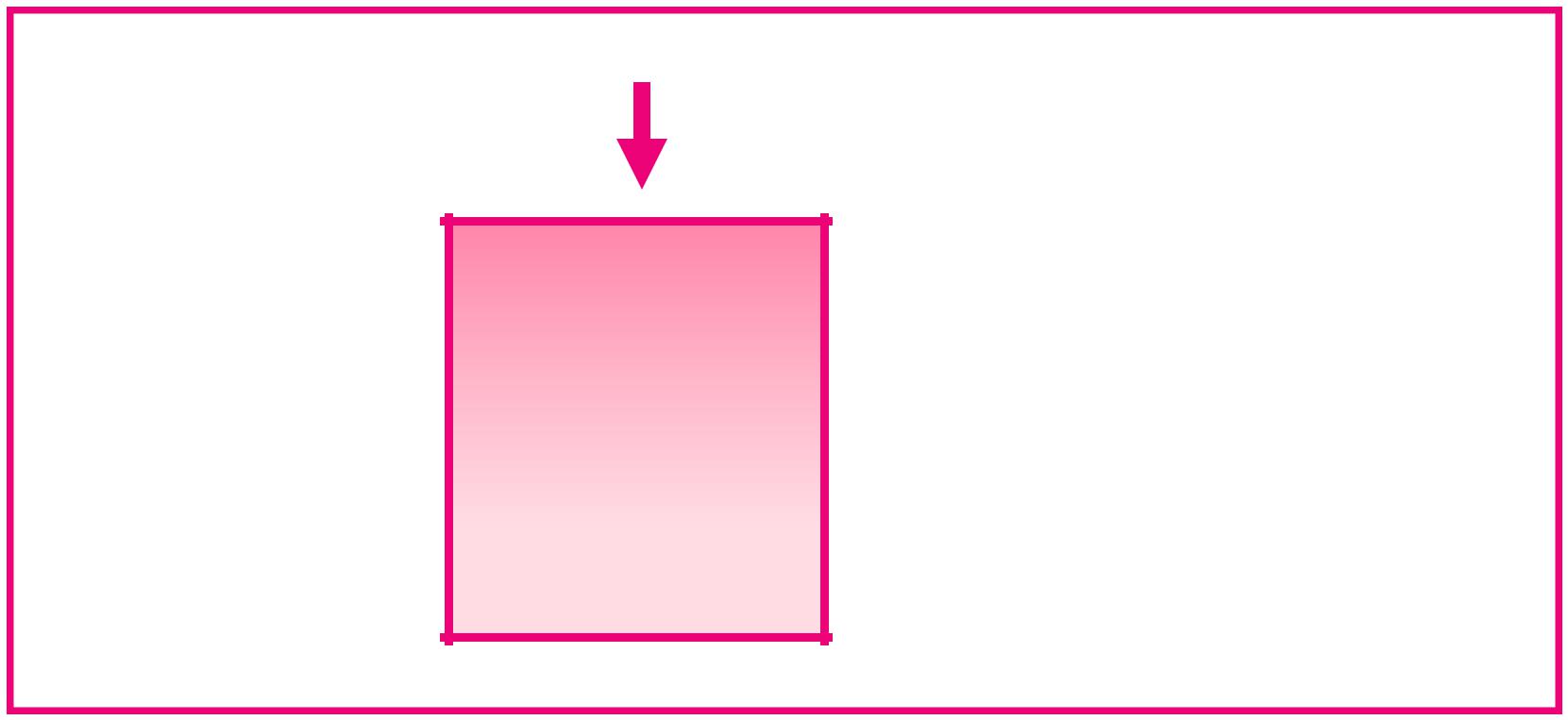
**YOUR RISK**

| **High** | **Above average (moderate)** | **Average** | **Low** |
| --- | --- | --- | --- |

| **80 to 92% of these**  **women will** | **93 to 95% of these**  **women will** | **96 to 98% of these**  **women will** | **More than 98% of these**  **women will** |
| --- | --- | --- | --- |
| **NOT develop breast** | **NOT develop breast** | **NOT develop breast** | **NOT develop breast** |
| **cancer.** | **cancer.** | **cancer.** | **cancer.** |
| 8 to 20% of these women will | 5 to 7% of these women will | 2 to 4% of these women | Less than 2% of these women will |
| develop the disease. | develop the disease. | will develop the disease. | develop the disease. |

Based on your questionnaire answers and mammogram, the following may have increased your risk of breast cancer:

- **[INSERT RISK FACTOR 1]**
- **[INSERT RISK FACTOR 2]**
- **[INSERT RISK FACTOR 3]**

However, these factor(s) may have helped to prevent your risk from being any higher:

- **[INSERT PREVENTATIVE FACTOR 1]**
- **[INSERT PREVENTATIVE FACTOR 2]**
- **[INSERT PREVENTATIVE FACTOR 3]**

Your risk of breast cancer can be reduced by up to 25% by making positive lifestyle changes, such as; adopting a healthy diet, taking regular exercise and losing weight (if needed). Such changes can also help reduce your risk of developing other diseases, such as heart disease, diabetes and dementia. More information on the ways to reduce your risk, together with the signs and symptoms of breast cancer are provided in the accompanying leaflet.

**We would encourage you to make an appointment with a doctor or nurse to discuss your risk further (Telephone: number). We can arrange either a telephone consultation or face-to-face appointment at [INSERT BUILDING NAME AND HOSPITAL NAME].** During this appointment, your breast cancer risk will be explained in more detail and further information will be provided on how to reduce your risk. For example, you may be eligible to take preventative medication or offered more frequent screening (invited to mammograms more frequently than 3-yearly).

Please remember that even though you have an increased risk of developing breast cancer in the next 10 years, **93 to 95%** **of women in your risk group will NOT develop breast cancer.**

Should you have any questions please get in touch with the BC-Predict Study team on **[telephone number]**, Monday to Friday 10am to 2pm.

Yours sincerely,

| Dear Ms Tester, |  |
| --- | --- |
| **RE: Breast Cancer (BC)-Predict Study** |  |
| **IMPORTANT: ALL CLEAR after your recent mammogram. This is NOT a recall.** |  |

Thank-you for taking part in the BC-Predict study. As part of that study we asked you to complete a questionnaire before you attended your mammogram. In this questionnaire you gave us information which enabled us to work out your risk of developing breast cancer in the next 10 years. Your risk is calculated from a combination of factors associated with your family history, lifestyle and breast density (the amount of tissue in your breast that is not fat).

Your risk of developing breast cancer in the next 10 years was calculated to be **high risk.** This means that 8 - 20% of women in your risk category will develop breast cancer **within the next 10 years.**

The table below provides you with information about your risk category and where you are in relation to other women:


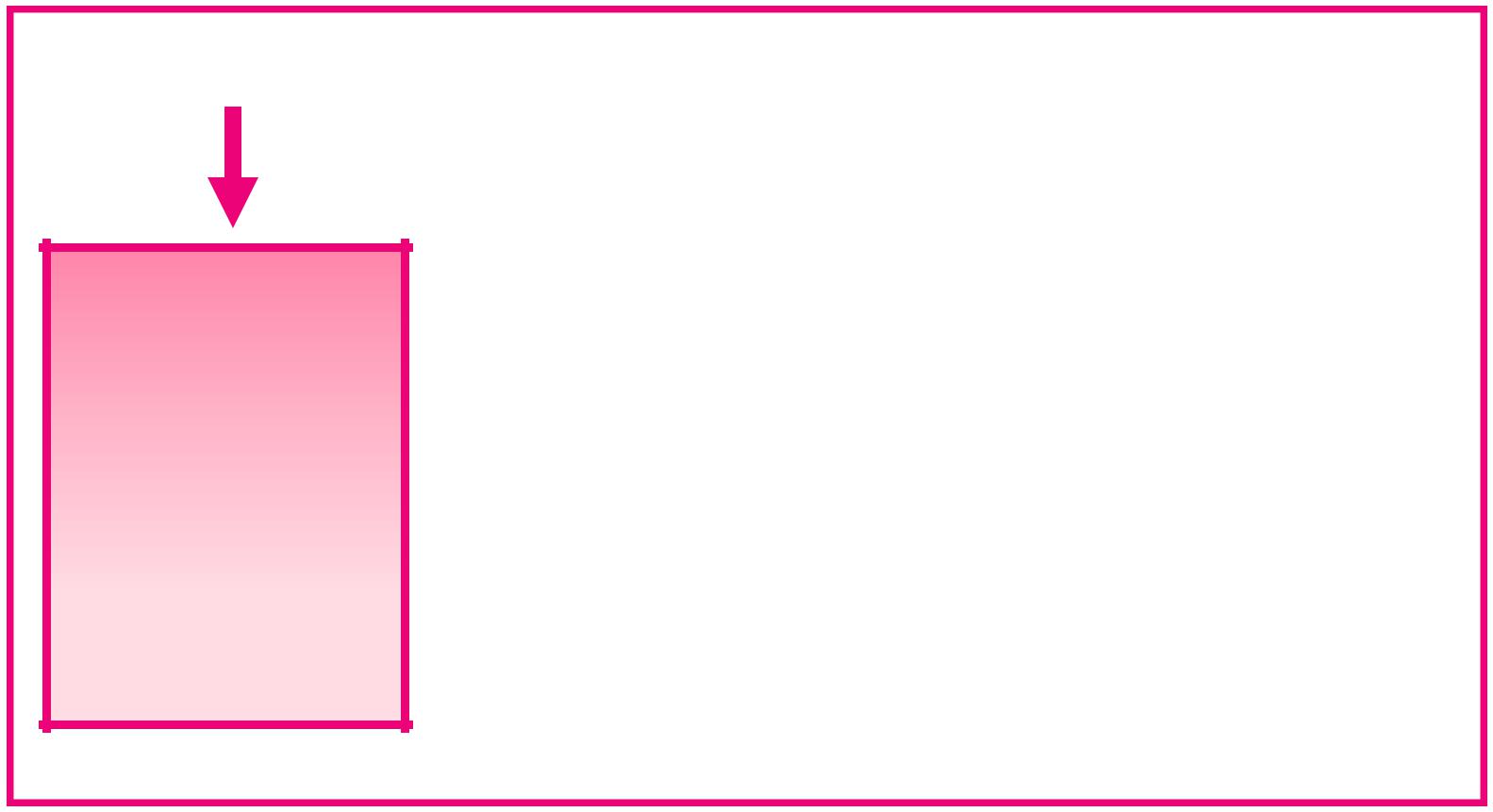
**YOUR RISK**

| **High** | **Above average (moderate)** | **Average** | **Low** |
| --- | --- | --- | --- |

| **80 to 92% of**  **these women** | **93 to 95% of these**  **women will** | **96 to 98% of these**  **women** | **More than 98% of these**  **women will** |
| --- | --- | --- | --- |
| **will NOT develop** | **NOT develop breast** | **will NOT develop** | **NOT develop breast** |
| **breast cancer.** | **cancer.** | **breast cancer.** | **cancer.** |
| 8 to 20% of these women | 5 to 7% of these women will | 2 to 4% of these women | Less than 2% of these women will |
| will develop the disease. | develop the disease. | will develop the disease. | develop the disease. |

Based on your questionnaire answers and mammogram, the following may have increased your risk of breast cancer:

- **[INSERT RISK FACTOR 1]**
- **[INSERT RISK FACTOR 2]**
- **[INSERT RISK FACTOR 3]**

However, these factor(s) may have helped to prevent your risk from being any higher:

- **[INSERT PREVENTATIVE FACTOR 1]**
- **[INSERT PREVENTATIVE FACTOR 2]**
- **[INSERT PREVENTATIVE FACTOR 3]**

Your risk of breast cancer can be reduced by up to 25% by making positive lifestyle changes, such as; adopting a healthy diet, taking regular exercise and losing weight (if needed). Such changes can also help reduce your risk of developing other diseases, such as heart disease, diabetes and dementia. More information on the ways to reduce your risk, together with the signs and symptoms of breast cancer are provided in the accompanying leaflet.

**We would encourage you to make an appointment with a doctor or nurse to discuss your risk further (Telephone: 0161 291 4408). We can arrange either a telephone consultation or face-to-face appointment at [INSERT BUILDING NAME AND HOSPITAL NAME].** During this appointment, your breast cancer risk will be explained in more detail and further information will be provided on how to reduce your risk. For example, you may be eligible to take preventative medication or offered more frequent screening (invited to mammograms more frequently than 3-yearly).

Please remember that even though you have an increased risk of developing breast cancer in the next 10 years, **80 to 92%** **of women in your risk group will NOT develop breast cancer.**

Should you have any questions please get in touch with the BC-Predict Study team on **[telephone number]**, Monday to Friday 10am to 2pm.

Yours sincerely,

1. **BC-Predict leaflet text (included with risk feedback letter)**

**This leaflet is designed to accompany your risk feedback letter**

**The BC-Predict Study**

You recently took part in a research study run by the Prevent Breast Cancer Research Unit and Nightingale Centre at Manchester University NHS Foundation Trust (MFT). This study is called BC-Predict.

BC-Predict aims to assess whether providing women with their estimated risk of developing breast cancer is feasible within the current NHS Breast Screening Programme. For this, we assessed your risk of developing breast cancer in the next 10 years.

**Background to breast cancer**

Breast cancer is the most common type of cancer in the UK.

In 2016, 55,122 women were diagnosed with breast cancer from the age of 20. Of these, 78% of women (3 out of 4) are predicted to live for 10 or more years after diagnosis.

This high rate of survival is in part due to cancers being detected at an earlier stage. The earlier a cancer is detected, the more effectively it can be treated.

**Breast cancer risk factors**

Many factors contribute to your risk of developing breast cancer. Some of these are modifiable and some are non-modifiable:

**Modifiable risk factors:**

- Being overweight
- Drinking more than 14 units of alcohol a week
- Being physically inactive – such as doing less than 2.5 hours of exercise a week
- Smoking

**Non-modifiable risk factors:**

- Age
- Family history
- Never having had children
- Being 30 years or older at the birth of your first child
- Starting your periods before the age of 12
- Going through the menopause after the age of 55

More detail about these factors can be found on the following page. Your risk has been calculated based on these factors.

**Non-modifiable risk factors**

**Age**

The older you are the greater your chances of developing breast cancer. Approximately 4 out of 5 breast cancers occur in women aged 50 and over.

**Family history**

Women who have male or female relatives diagnosed with breast cancer have a higher risk of developing the disease themselves.

- Risk increases with the number of close relatives diagnosed, especially if they were diagnosed at a younger age.
- But even so, approximately 8 out of 10 breast cancers occur in women with no close relatives diagnosed with the disease.

**Breast density**

Breasts are made up of glandular tissue, connective tissue and fat. Women with dense breasts have more glandular and connective tissue than fat. The risk of breast cancer is higher in women with dense breasts because there is more tissue that could potentially become cancerous. How dense your breasts are varies greatly between women but can be inherited from female family members such as your Mother. Dense breast tissue can make a mammogram more difficult to read because abnormal lumps are harder to detect.

**Hormones and reproduction**

The female sex hormones, oestrogen and progesterone, can affect the development of breast cancer.

- Hormone Replacement Therapy (HRT) increases the risk of breast cancer. The risk associated with HRT is reduced 5 years after you stop taking it.
- Oral contraceptives (also known as the Pill) increase the risk of breast cancer but can reduce the risk of ovarian and womb cancers. The risk associated with the Pill is reduced 10 years after you stop taking it.
- Starting your periods at a younger age or having a late menopause increases the risk of breast cancer. This is due to being exposed to the female sex hormones for longer.
- Having children and breast feeding both lower the chances of developing the disease.

**Modifiable risk factors**

**Maintain a healthy weight**

Being overweight and gaining weight throughout adult life increases the risk of developing breast cancer after the menopause. Try to maintain a healthy weight by combining a balanced diet, including plenty of fruit and vegetables, with regular physical activity. For advice on how to maintain a healthy weight, please see the NHS Choices website at: [www.nhs.uk/live-well/eat-well/](http://www.nhs.uk/live-well/eat-well/)

**Physical activity**

Women who are physically active are less likely to develop breast cancer than non-active women. Try to do at least 2.5 hours of moderate physical activity a week, such as 30 minutes of brisk walking five times a week and strength exercises at least twice a week. For advice on how to increase physical activity, consult your GP practice or see the NHS Choices website at: [www.nhs.uk/live-well/exercise/free-fitness-ideas/](http://www.nhs.uk/live-well/exercise/free-fitness-ideas/)

**Limiting alcohol**

Drinking alcohol raises the risk of breast cancer. Try to keep to sensible intakes of less than 14 units a week, or 2-3 units a day ensuring you have at least 2 alcohol free days a week.

- 14 units is around a bottle and a half of wine a week or 10 measures of spirit a week.
- 2-3 units a day of alcohol is approximately 1 medium-large glass of wine, a pint of beer or 2 measures of spirits. For advice on reducing your alcohol intake, please see the NHS Choices website at: [www.nhs.uk/live-well/alcohol-support/](http://www.nhs.uk/live-well/alcohol-support/)

**Not smoking**

As well as increasing your risk of developing heart disease and lung cancer there is also evidence to suggest that smoking increases your risk of developing breast cancer. For advice on how to stop smoking, contact your GP or join an NHS Stop Smoking Service. You can find your local service through the website [www.nhs.uk/smokefree](http://www.nhs.uk/smokefree) or call the Smokefree National Helpline to speak to a trained adviser on 0800 0224 332.

**Preventative medication**

Together with maintaining a healthy lifestyle, women at **above average (moderate)** and **high risk** may also be eligible to take a drug for prevention, please see your accompanying risk letter for more details.

**Signs and symptoms of breast cancer**

If you get to know how your breasts normally look and feel, you will be more likely to spot any changes that could be signs of breast cancer. This is important, even if you have been for breast screening. Look out for the following:

- A lump or thickening in the breast
- A change in the nipple. The nipple might be pulled back into the breast, or change shape. You might have a rash that makes the nipple look red and scaly, or have blood or another fluid coming from the nipple.
- A change in how your breasts feel or look. They may feel heavy, warm or uneven, or the skin may look dimpled. The size and shape of the breast may change.
- Pain or discomfort in the breast or armpit.
- A swelling or lump in the arm

Even women with a below average risk can still develop breast cancer, so it is important to know the signs of breast cancer and what you can do to reduce your risk.

If you have any changes to your breasts, you should make an appointment to see your GP straight away. It is unlikely that you have breast cancer, but if you do, being diagnosed and treated at an early stage makes it more likely that you can be successfully treated.

If you have a family history of breast cancer you should discuss this with your GP who may refer you to a Family History Clinic (FHC) where you can discuss your risk further.

If you choose to do so, talking about and sharing any concerns you may have about your risk of developing breast cancer with your loved ones may help to reduce some of your worries.

If you have any questions about your routine breast screening appointments, you can contact your GP or your local breast screening service. Please find the contact details at the end of your risk letter.

You may find the following websites helpful to learn more about breast cancer and ways to reduce your risk:

**Prevent Breast Cancer: www.preventbreastcancer.org.uk**

**Cancer Research UK: www.cancerresearchuk.org**

**Macmillan Cancer Support: www.macmillan.org.uk**

**Breast Cancer Care UK**: **www.breastcancercare.org.uk**

**Breast Cancer Now**: **www.breastcancernow.org**

For further details regarding the BC-Predict study, please contact [study team contact details]

1. **Researcher reflexivity statement**

IPA researchers require an awareness of their positionality and influence during the research process. This study forms part of a PhD exploring breast cancer risk appraisals following a clinically-derived risk estimate and is informed by previous research conducted by the authors, who are members of the BC-Predict team and have conducted a large body of research on implementing population-level risk-stratified screening. This may have impacted how the data were interpreted. However, prior to conducting the research the primary author aimed to bracket these pre-existing thoughts influenced by previous research and identified potential biases by listing these before interviewing. After each interview the primary author produced reflexive notes to capture initial thoughts about interviewee’s experiences to facilitate analysis. Although the analysis was carried out by the primary author, other members of the research team with expertise in qualitative research challenged perceptions which may have been influenced by *a priori*  ideas. On a personal level, the primary author has never had any immediate experiences of breast cancer nor, due to her age, attended breast screening which may have influenced interpretations of women’s experiences. Nevertheless, the primary author has extensive experience of working with this population, so it is unlikely a lack of personal experience had any significant impact on the analysis presented.

1. **PETs tables per participant**

**Ann**

| **Personal experiential theme title** | **Experiential statements** |
| --- | --- |
| Given capacity to consider breast cancer | *Affected relative acting as a gate keeper to the realities of breast cancer*  *Not in control of considering the significance of breast cancer – shielded*  *Stoical personalities of affected women influencing personal significance attributed to breast cancer*  *Breast cancer not fatal (not cause of death)*  *Age and competing personal priorities vs accessing significance of breast cancer* |
| Internal conflict in defining causal attributions for breast cancer | *Unable to align ‘healthy living’ with breast cancer prevention*  *Internal model of a women with BC vs physical instances – incongruent (health)*  *Lacks confidence in defining breast cancer cause and effect*  *Breast cancer does not discriminate*  *Breast cancer is random*  *Conflict over choice and control over breast cancer onset*  *Trajectory unpredictable* |
| Futility of reflecting on behaviours of past self | *Damage of past health behaviours unavoidable in the present*  *Powerless to control past self*  *Duty to self in the present*  *Absolved personal responsibility of past self*  *Doesn’t do to dwell* |
| Risk notification lacked personal impact | *Lack of confidence in recall exact risk result*  *Unsurprised by result, expectations reflected appraisals*  *Consultation made little lasting impression*  *Risk notification is not diagnostic*  *Perseveration of mental health priority*  *Doesn’t do to dwell or identify too deeply with risk* |

**Jill**

| **Personal experiential theme title** | **Experiential statements** |
| --- | --- |
| Limited exposure to breast cancer and affected relative’s journey | *Physically distance from affected relative*  *Non-active care role for affected relative*  *Vague knowledge of Aunt’s breast cancer*  *Relationship define as not a particularly close one* |
| Disassociating/disconnecting with breast cancer | *Cause of death in relative more significant than breast cancer diagnosis (dementia)*  *Breast cancer not in her life plan (not destined to develop)*  *Does not associate with the cancers in her family*  *Personal breast lump journey, yet breast cancer not meant for her*  *Breast lump did not invoke thoughts of a diagnosis*  *Breast cancer pre-determined for some, but not for her* |
| Difficulties in defining a concrete pattern for a breast cancer diagnosis | *Healthy living has protective value, or does it?*  *Breast cancer is pre-determined*  *Breast cancer is random and does not discriminate*  *Unhealthy women get breast cancer?*  *Genetically predisposed?* |
| Clinically-derived risk taken at face value | *Risk as reasonable – not thought of too closely*  *Identifies with those who live healthily not necessarily those at increased’ risk*  *Risk made no emotional impact*  *Hierarchy of disease worry employed – breast cancer not necessarily near the top*  *Self-preservation and mental health valued more* |
| Risk notification an encouraging reminder for continued engagement in positive health behaviours | *Encouraged breast awareness and vigilance*  *Personal responsibility to remain ‘healthy’* |

**Michelle**

| **Personal experiential theme titles** | **Personal experiential statements** |
| --- | --- |
| Breast cancer a strong identifying feature in family life | *Breast cancer as a constant companion*  *Breast cancer is a shared family experience*  *Collective concern within the family*  *Breast cancer is a defining feature in family life*  *Breast cancer bonds the family*  *Rely on family members to discuss and support*  *Openness in the family helps manage the threat*  *Family communication valued* |
| Breast cancer a natural part of life | *Breast cancer so natural does not require conscious thought*  *Risk is there but doesn’t invade consciousness*  *Breast cancer a natural feature of life* |
| No definitive reasons for a breast cancer diagnosis | *Breast cancer is random*  *Breast cancer doesn’t discriminate*  *No blame attributable to those diagnosed*  *No pattern to breast cancer*  *Breast cancer is inevitable for some – no control, no preventatble* |
| Personal risk attributable to a genetic predisposition? | *Expectation of risk based on family history links*  *Feels genetically predisposed*  *Appraisal of risk based on level of family history*  *A ‘genetic plan’ – predetermined*  *Breast cancer potentially non-preventable due to genetics* |
| Risk notification caused an unexpected journey | *Risk caused shock despite family history knowledge BUT expectations met*  *Risk notification as a wake-up call, sit up and take notice*  *Offer of prevention drug caused shock*  *Unknowingly embarked on a new risk journey*  *Needing to be physical engaged in the risk notification context to place it in her life*  *Risk needed time to process* |
| Careful deliberation of Tamoxifen use | *Risk prevention drug considered in the context of current health concerns (menopause and HRT use)*  *Managing menopausal symptoms current priority*  *Quality of life concerns if remove HRT*  *Deep thought required regarding tamoxifen use*  *Natural HRT considered carefully* |

**Sue**

| **Personal experiential theme titles** | **Personal experiential statements** |
| --- | --- |
| Limited ability to support others with cancer | *Limited exposure to breast cancer in others*  *Physically distanced from affected friend*  *Physically distanced from father’s cancer diagnosis*  *Own priorities took precedence* |
| Personal autonomy and control over health behaviours | *Empowered by own autonomy over lifestyle*  *Conflict over level of control if genetically predisposed*  *Risk notification is an opportunity to change, a diagnosis is final*  *Dubious about women’s health information* |
| Personal risk perplexing | *Contributors to risk estimate perplexing*  *Cannot understand by certain risk factors contributed to an increase risk*  *Risk notification unexpected*  *No reason to disbelieve but not convinced*  *Conflict in self with identifying with the risk estimate.* |
| Unhelpful to dwell on breast cancer risk | *Cannot dwell on uncertainties*  *Futile to use years to worrying*  *Breast cancer is random* |

**Lindsey**

| **Personal experiential theme titles** | **Personal experiential statements** |
| --- | --- |
| Limited personal exposure to breast cancer | *Breast cancer does not feature in life*  *Cancer not a defining feature of family life*  *Breast cancer is not front of mind*  *Direct experience said to perhaps influence her awareness more*  *Breast cancer has not ‘touched’ her life*  *Breast cancer not thought with regularity*  *Susceptibility not considered often* |
| Difficulty aligning ‘healthy living’ with a breast cancer diagnosis | *Positive health behaviours should have protective value*  *Difficulty aligning health behaviours with a breast cancer diagnosis*  *Healthy women get breast cancer but some unhealthy women don’t*  *A breast cancer diagnosis should be for those typically unhealthy?* |
| No rhyme or reason to breast cancer | *Breast cancer is ultimately random*  *Breast cancer is unpredictable*  *Breast cancer diagnosis is bad luck*  *Genetic ‘elements’ can contribute*  *No pattern to getting breast cancer* |
| Accepts but does not identify with risk | *Risk notification caused surprise*  *Cannot legitimise surprise as had no preconceived ideas of personal risk*  *Lack of family history indicator of not identifying as increased risk*  *Lifestyle does not fit with someone at ‘increased risk’*  *Risk notification not distressing*  *Does not identify with her version of someone at increased risk*  *Contributing risk factors suprising* |
| Risk notification caused a state of prevention limbo | *Confusion as to what more can be done to reduce risk*  *Helpless state that she cannot do anymore regarding her health behaviours*  *Defeatist – cannot alter risk*  *A limit of what she can do to prevent breast cancer*  *No answers on how to reduce risk*  *Does not identify as someone eligible for preventative medication* |

**Bev**

| **Personal experiential theme titles** | **Personal experiential statements** |
| --- | --- |
| Breast cancer is not a death sentence | *Breast cancer is treatable*  *Colleague successful overcame breast cancer*  *Breast cancer not the biggest disease concern*  *A ‘normal’ life is possible after a diagnosis* |
| Difficulty aligning ‘health behaviours’ with women diagnosed with breast cancer | *Colleague not a ‘typical’ example of a woman with breast cancer*  *Colleague not a ‘candidate’ for the disease*  *Women with breast cancer have unhealthy lifestyles?*  *Breast cancer ought to happen to those with multiple risk factors* |
| Maintaining at state of nativity over breast cancer | *Favours a state of nativity over breast cancer*  *Better to preserve mental health*  *Purposefully avoids thinking about breast cancer risk*  *Chooses not to dwell on breast cancer risk* |
| Breast cancer not in her future | *Breast cancer diagnosis unlikely*  *Lack of family history has protective value*  *Does not identify with main breast cancer risk factors*  *Does not identify with her ideas of a woman at increased risk/diagnosed with breast cancer* |
| Maintaining a low risk appraisal | *Identifies as ‘lowish risk’*  *Perceives self to be below population risk*  *Inaccurate recall of actual risk estimate*  *Risk notification made no lasting impression*  *Unperturbed/unfazed by clinical risk*  *Does not identify with ‘risky behaviours’*  *Not a candidate for preventative medication* |
| Breast cancer risk not a personal priority | *One risk among many*  *More to life than disease risk*  *Breast cancer risk doesn’t feature highly on list of health concerns*  *Breast cancer risk should not be considered in isolation of other health issues*  *Preservation of mental wellbeing takes priority* |

**Yvonne**

| **Personal experiential theme titles** | **Personal experiential statements** |
| --- | --- |
| Cruelty of cancer inflicting those who are young | *Diagnosis of cancer at young age hits differently*  *Confusion as to why breast cancer can happen in young women*  *Breast cancer should be an older woman’s disease* |
| Cancer is fated | *Cancer is sneaky*  *Cancer will seek you out, if it is in your life plan*  *Cancer is already written*  *Cancer cannot be controlled or stopped* |
| A lack of family history is protective | *Breast cancer not a priority as no family history*  *Lack of family history drives a low risk appraisal*  *Breast cancer risk not considered closely* |
| Unperturbed by risk notification | *Inaccurate recall of risk notification*  *Risk notification made not lasting impression*  *No need to make lifestyle changes*  *Risk appraisal unaltered by risk notification* |

**Dawn**

| **Personal experiential theme titles** | **Personal experiential statements** |
| --- | --- |
| Overwhelming experiences of mother’s breast cancer | *Intrusive memories define family life*  *Emotional toll of mother’s breast cancer still felt*  *Trauma from mother’s breast cancer required recovery time*  *Anxiety peaked when reaching the age of mother’s death*  *Anxiety transferred to daughter and granddaughter* |
| Overwhelming sense of duty toward mother’s care | *Very active care role throughout mother’s breast cancer diagnosis*  *Internal pressure to care and protect*  *Physically exhausted by care duties*  *Mother’s care permeated all areas of life* |
| Breast cancer does not discriminate | *No fairness in who is ‘chosen’ to develop breast cancer*  *Breast cancer is random, no patterns or reasons*  *Conflict between ‘unhealthy’ living and ‘healthy’ women developing breast cancer* |
| External factors associated with mother’s breast cancer | *Mother’s breast cancer caused by external stresses*  *Mother had ‘difficult’ life – contributed to breast cancer*  *Genetic causes of mother’s cancer an after-thought* |
| Risk notification a call to action | *Risk notification led to ways to reduce/prevent cancer risk*  *Risk notification enabled control*  *Did not hesitate to reduce risk with medication*  *Risk notification awakened awareness* |
| Risk notification needed to time to process | *Time aids acceptance of risk*  *Risk expected but still caused shock*  *Physical notification made risk real* |
| Breast cancer not the only health priority | *Breast cancer not thought in isolation of other illnesses*  *Breast cancer competes for ‘top spot’ in illness hierarchy*  *Other immediate health concerns require attention*  *Breast cancer is in the background* |

1. **IPA quality evaluation guide, based on Smith, 2011*.**

| **Item No.** | **Description/Criterion** | **Location in the manuscript** |
| --- | --- | --- |
| 1 | Clearly subscribes to the theoretical principles of IPA: it is phenomenological, hermeneutic and idiographic. | Pg. 3-14 & supplementary file. |
| 2 | Sufficiently transparent so reader can see what was done. | Pg. 3-4 |
| 3 | Coherent, plausible and interesting analysis. | Pg. 5-14 |
| 4 | Sufficient sampling from corpus to show density of evidence for each theme:   - N1-3: extracts from every participant for each theme; - **N4-8: extracts from at least three participants for each theme;** - N>8: extracts from at least three participants for each theme+measure of prevalence of themes, or extracts from half the sample for each theme. | Pg. 5-14 |
| 5 | ***The paper should have a clear focus.*** Papers providing detail of a particular aspect rather than abroad reconnaissance are more likely to be of high quality. This focus may be determined at the outset or emerge during analysis. | Throughout Introduction & results |
| 6 | ***The paper will have strong data.*** Most IPA is derived from interviews and this means that, for the most part, getting good data requires doing good interviewing. This particular skill must not be underestimated. The quality of the interview data obtained sets a cap on how good a paper can subsequently be. | Pg. 4-14 |
| 7 | ***The paper should be rigorous.*** One should aim to give some measure of prevalence for a theme and the corpus should be well represented in the analysis. Extracts should be selected to give some indication of convergence and divergence, representativeness and variability. This way the reader gets to see the breadth and depth of the theme. For papers with small sample sizes (1-3), each theme should be supported with extracts from each participant. **For papers with sample sizes of 4-8, in general, extracts from half the participants should be provided as evidence.** For larger sample sizes, researchers should give illustrations from at least three or four participants per theme and also provide some indication of how prevalence of a theme is determined. | Pg. 5-14 |
| 8 | ***Sufficient space must be given to the elaboration of each theme.*** In certain circumstances it may well be better to present a subset of the emergent themes so there is room to do justice to each, rather than presenting all themes but doing so superficially. | Pg. 5-14 |
| 9 | ***The analysis should be pointing to both convergence and divergence.*** Where an IPA study reports data from more than one participant, there should be a skilful demonstration of both patterns of similarity among participants as well as the uniqueness of the individual experience. The unfolding narrative for a theme thus provides a careful interpretative analysis of how participants manifest the same theme in particular and different ways. This nuanced capturing of similarity and difference, convergence and divergence is the hallmark of good IPA work. | Pg. 5-14 |
| 10 | ***The paper needs to be carefully written.*** Good qualitative work always requires good writing. The reader will feel engaged by a well-wrought, sustained narrative. As a result, he/she will consider they have learned in detail about the participants’ experience of the phenomenon under investigation | Throughout the manuscript & supplementary file. |

* Smith, J. A. (2011). Evaluating the contribution of interpretative phenomenological analysis. Health psychology review, 5(1), 9-27.
